# Cardiac Non-Human Leukocyte Antigen Identification: Techniques and Troubles

**DOI:** 10.3389/fimmu.2017.01332

**Published:** 2017-10-18

**Authors:** Katherine V. Gates, Naveen L. Pereira, Leigh G. Griffiths

**Affiliations:** ^1^Department of Veterinary Medicine and Epidemiology, University of California, Davis, Davis, CA, United States; ^2^Department of Cardiovascular Diseases, Mayo Clinic, Rochester, MN, United States

**Keywords:** proteomics, immunoproteomics, non-human leukocyte antigen antibody, antigen identification, heart transplantation

## Abstract

Historically efforts have focused on the human leukocyte antigen (HLA) as the major cause for acute and chronic rejection following cardiac transplantation. However, rising evidence indicates that non-HLA antibodies can be both primary initiators and modifiers of antibody-mediated rejection (AMR) and cardiac allograft vasculopathy (CAV). The purpose of this review is to assess currently available technologies for non-HLA identification and leveraging such responses toward antibody quantification. Several techniques have been used to identify antigenic determinants of recipient graft-specific non-HLA humoral immune responses, but each comes with its own set of benefits and caveats. Improving our ability to detect non-HLA humoral immune response will aid in our understanding of the underlying antigenic determinants of AMR and CAV, as well as improve patient outcomes.

## Introduction

Since the first cardiac allograft transplantation in 1967 (1), advances in diagnosis and treatment of acute rejection have led to dramatic improvements in recipient survival (1). Despite advances in management of acute rejection episodes, rate of chronic allograft dysfunction and rejection have remained largely unchanged (1, 2). Furthermore, chronic immune system activation has been implicated as an important contributor to chronic allograft dysfunction and cardiac allograft vasculopathy (CAV) (3). CAV results in 30% of all postcardiac transplantation deaths beyond 1 year postoperatively (4). Consequently, although modern immunosuppressant regimens have alleviated most acute rejection episodes, these drugs fail to fully control less well understood chronic rejection processes. Enhanced understanding of the mechanisms leading to chronic rejection, allograft dysfunction, and CAV is needed to further enhance survival for cardiac transplant recipients.

Endomyocardial biopsy (EMB) represents the current gold standard method for monitoring rejection episodes. However, this technique is highly invasive, with current International Society of Heart Lung Transplantation (ISHLT) guidelines recommending a total of 16 biopsies in the first-year posttransplant (5). After 1 year, EMB is recommended only once the patient is exhibiting symptoms, or signs of graft dysfunction such as fatigue, nausea, fever, dyspnea, edema, and arrhythmias (5). However, even with optimal postoperative follow-up, this invasive monitoring strategy fails to fully characterize the underlying causes of graft-dysfunction, or predict impending acute or chronic rejection. Furthermore, although several imaging technologies (e.g., angiography, optical coherence tomography, intravascular ultrasound, positron emission tomography) have proven useful for monitoring heart transplant recipients and follow CAV progression, such modalities lack the ability to identify the underlying cause of rejection and pathogenesis of CAV (6–10). The shortcomings of current transplant rejection monitoring strategies have stimulated intense interest in defining the primary determinants of acute and chronic cardiac rejection, and leveraging such knowledge toward development of biomarker screening assays for non-invasive assessment of rejection episodes to improve long-term outcomes.

Rejection episodes have been broadly defined in three categories, hyperacute, acute, and chronic. Hyperacute rejection occurs when preformed antibodies toward the transplanted organ resulting in activation of the complement cascade, massive coagulation, and organ loss. The existence of preformed graft-specific antibodies results in essentially immediate activation of this process following perfusion of the organ with the recipient’s circulation. Consequently, the time frame for hyperacute rejection episodes is on the order of minutes to hours (11). This type of rejection is rare due to routine screening using ABO blood group matching, virtual human leukocyte antigen (HLA) crossmatch tests (12) and panel reactive antibodies (13) to ensure that preformed antidonor antibodies are not present in the recipients circulation.

Risk for acute rejection episodes begins within the first few weeks posttransplant, but can be seen as far out as several years. It falls broadly into two categories, acute cellular rejection (ACR) and antibody-mediated rejection (AMR) (3), referring to a response that primarily involves the cell-mediated or humoral arm of the immune system, respectively. However, it should be noted that AMR and ACR are not entirely disparate mechanisms, as IgG antibody production is in part mediated by CD4+ T-helper cell help and cell-mediated responses are commonly modulated by antibody deposition (14). The diagnosis of ACR is generally made by visualizing lymphocyte and/or macrophage infiltrates, with or without cardiomyocyte necrosis on EMB (5). The mechanism behind ACR is a T-cell dominated immune attack against the myocardium that could result in necrosis and organ failure (3). Such infiltrating T-cells commonly respond to the highly polymorphic nature of HLA *via* direct or indirect pathways (15, 16). In the direct pathway, recipient T-cells recognize foreign HLA on donor antigen-presenting cells (APCs), while the indirect pathway results from recipient T-cells recognizing donor-derived peptides presented by recipient APCs (17). It is thought that the direct pathway will decline with time due to the eventual loss of donor APCs, while the smaller pool of indirectly activated T-cells will grow with time due to clonal expansion (18). AMR is less well understood, although the prevailing view currently implicates antibodies directed against HLA and/or non-HLAs leading to complement activation and organ dysfunction (19). AMR is diagnosed *via* EMB by identifying classic histologic changes and antibody binding, with resultant complement deposition, through assessment of classical pathway activation, using C4d staining (19, 20). The advent of modern immunosuppressant regimens have reduced the incidence of acute rejection to 19% and allows for control of such episodes once the diagnosis is made (3). However, once a patient experiences an acute rejection episode, their risk of developing CAV and chronic rejection increases (4). Specifically, AMR patients have ninefold increased risk of developing CAV compared to ACR patients (2). Chronic rejection occurs several years posttransplant, predominantly manifesting as CAV leading to graft dysfunction and death. Although development of both HLA and non-HLA antibodies are clear contributing factors (21), the precise mechanisms responsible for the development of CAV remain elusive. This review will focus on techniques which utilize humoral immune responses to identify antigenic determinants responsible for AMR, chronic rejection and CAV.

### Recipient Immune Responses beyond HLA

**Figure 1:**
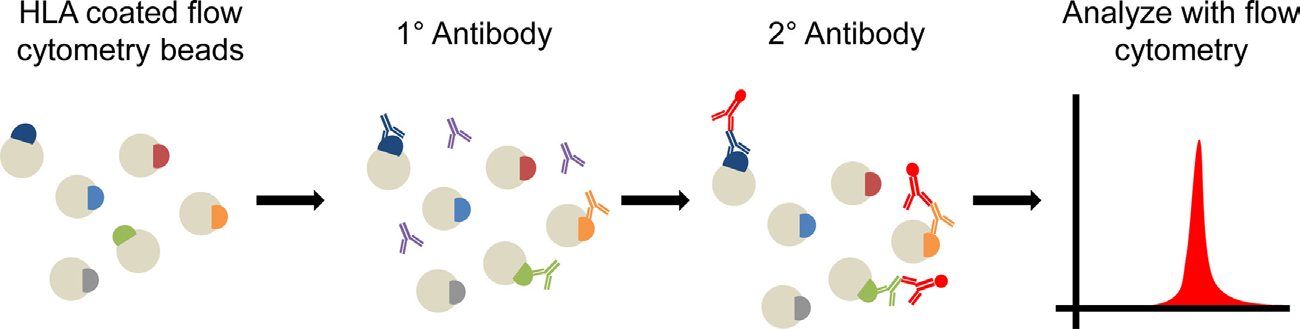
Diagram of solid phase cross matching. Flow cytometry beads are coated with human leukocyte antigen (HLA) and then probed with the patient’s serum. Recipient anti-HLA antibodies will bind to beads containing those HLA epitopes which the recipient immune system recognizes. A fluorscent antihuman secondary antibody then binds to the patient’s antibodies. Beads are run on a flow cytometer and the presence and number (mean fluorescence intensity) of HLA antibodies can be determined. Longitudinal assessment of antibody presence in an individual patient can be used to assess presence of preexisting anti-HLA antibodies and formation of *de novo* anti-HLA antibodies following transplantation.

Human leukocyte antigen (HLA) mismatch is the primary barrier to allograft transplantation, although recent studies (22–24) have highlighted the role of non-HLAs in initiating or modulating rejection responses. Current clinical recommendations rely heavily on pre- and postoperative assessment of anti-HLA antibodies for prediction and monitoring of rejection episodes (25). Significant improvements have been made in HLA-matching by the use of modern solid phase flow techniques such as single antigen bead (SAB) technology for virtual cross-match with HLA epitope matching (Figure 1) (25). However, HLA responses alone are insufficient to account for all deleterious immune responses observed in transplant recipients. Growing recognition of the importance of less polymorphic, but still antigenic non-HLA targets in transplant rejection has led to increased focus on such antigens in transplant immunology (22–24, 26). Recent evidence indicates that the interplay between HLA and non-HLA antibodies may accelerate both acute and chronic rejection (23). Most intriguingly, recent studies have identified a select cohort of transplant recipients who suffer from AMR without detectable donor-specific anti-HLA antibodies (DSA), indicating an antibody response dominated by non-HLA mechanisms (27). Such findings have resulted in calls for inclusion of assessment for non-HLA antibodies in routine screening of transplant recipients (24). While many experiments have indicated increased risk of transplant rejection in patients with the presence of non-HLA antibodies (23, 28), the relative contribution of individual non-HLAs to graft-specific immune responses remain largely unknown.

**Figure 2:**
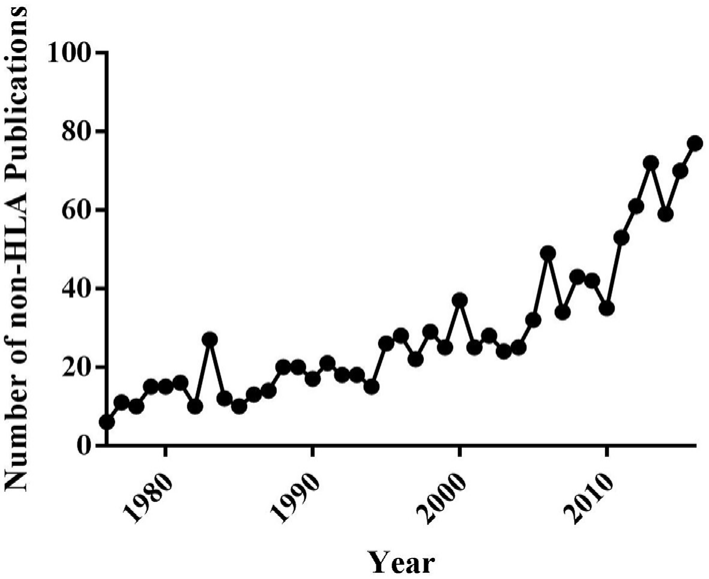
Timeline of the number of publications about non-human leukocyte antigen (non-HLA) in PubMed from years 1976 to 2016.

The emerging importance of non-HLA antibodies both as primary initiators and modifiers of AMR episodes, which potentiate chronic rejection and CAV, highlights the need for greater understanding of the specific identities of non-HLAs. The number of articles focusing on non-HLA antibodies has exploded in the last 20 years (Figure 2) (22, 24, 29–31), reflecting the increased appreciation for the importance of such antigens in graft-specific rejection responses. However, significant challenges remain to be overcome before a complete understanding of the identities, relative immunogenicity, and additive/synergistic contributions of non-HLA and HLA mediated responses in graft-specific rejection responses can be achieved. Finally, the ability of this information to be leveraged longitudinally for application as biomarkers of rejection remains to be determined. A logical first step in improving understanding of non-HLA-related rejection responses is to identify the spectrum of antigenic components involved in AMR, chronic rejection and CAV. Here, we will review (1) current important cardiac non-HLAs, (2) technologies available for non-HLA discovery, (3) limitations of current technologies, and (4) what the future holds for antigen discovery technology. This review will also focus specifically on cardiac transplants, but will expand to other transplants if a technology has only been applied to other solid organs.

## Historic Non-HLA Discovery Technologies

Immunoproteomic methods have come to the forefront of transplant immunology, due to their ability to identify the molecular targets of non-HLA antibodies. This type of technology has become popular in part because it allows for non-invasive identification of non-HLA targets in transplant recipients through blood sample analysis instead of biopsy. As the name suggests there are two basic components of immunoproteomics, the immuno—which refers to antibodies formed against antigenic epitopes and the—proteomics, which denotes the technology used to identify such antigens. The majority of current technologies utilize similar fundamental components of the immunoproteomic procedure: proteins are immobilized on a substrate that can be probed with patient serum, with reactive proteins isolated and identified. However, while at its core this process seems streamlined, several issues plague many of the current techniques. Here, we will discuss current immunoproteomic methods utilized for antigen identification and highlight the benefits and shortcomings of each technique.

### Non-Immunoproteomic Techniques

A small number of cardiac non-HLAs were initially identified using non-immunoproteomic techniques. Indeed, the discovery of the most extensively investigated known cardiac non-HLA proteins, MHC class I polypeptide-related sequence A (MICA) (32), angiotensin II type-1 receptor (AT_1_R) (33), and endothelin-1 type A receptor (ET_A_R) (34), was achieved through a combination of chance and hypothesis driven research. In 1994, the polymorphic structure of MICA was found to be similar to HLA (35), while in 2000 MICA was discovered on the surface of endothelial cells (30). Researchers hypothesized that the polymorphism of MICA combined with its vascular distribution made it a likely target for immune activation. Following development of a recombinant MICA enzyme-linked immunosorbent assay (ELISA), the hypothesis was supported by the finding that cardiac transplant recipients demonstrated elevated anti-MICA antibody titers (30). Similarly, in patients with hypertension or preeclampsia, immune responses toward AT_1_R had been extensively researched and implicated in renal dysfunction (36). Pathologic findings in hypertensive renal disease were similar to those observed in renal transplants, prompting investigation of AT_1_R as a potentially important antigen in renal transplantation. Assessment of antibody binding using surface-plasmon-resonance confirmed the presence of anti-AT_1_R antibodies in renal transplant recipients (36). Finally, since both AT_1_R and ET_A_R were implicated in endothelial activation, researchers investigated their role as non-HLA targets in cardiac transplant recipients. Assessment of anti-AT_1_R and ET_A_R antibody titers *via* ELISA confirmed that both receptors are antigenic in cardiac transplant recipients (34). However, in all three cases, whether the observed humoral response is due to a polymorphic alloantigen response or uncovering of an autoantigen remains unclear. Although traditional hypothesis driven research led to the identification of MICA, AT_1_R and ET_A_R as non-HLAs, application of proteomic methodologies to transplant immunology facilitates a hypothesis-generating, high-throughput approach to antigen identification.

### Two-Dimensional Gel Electrophoresis

**Figure 3:**
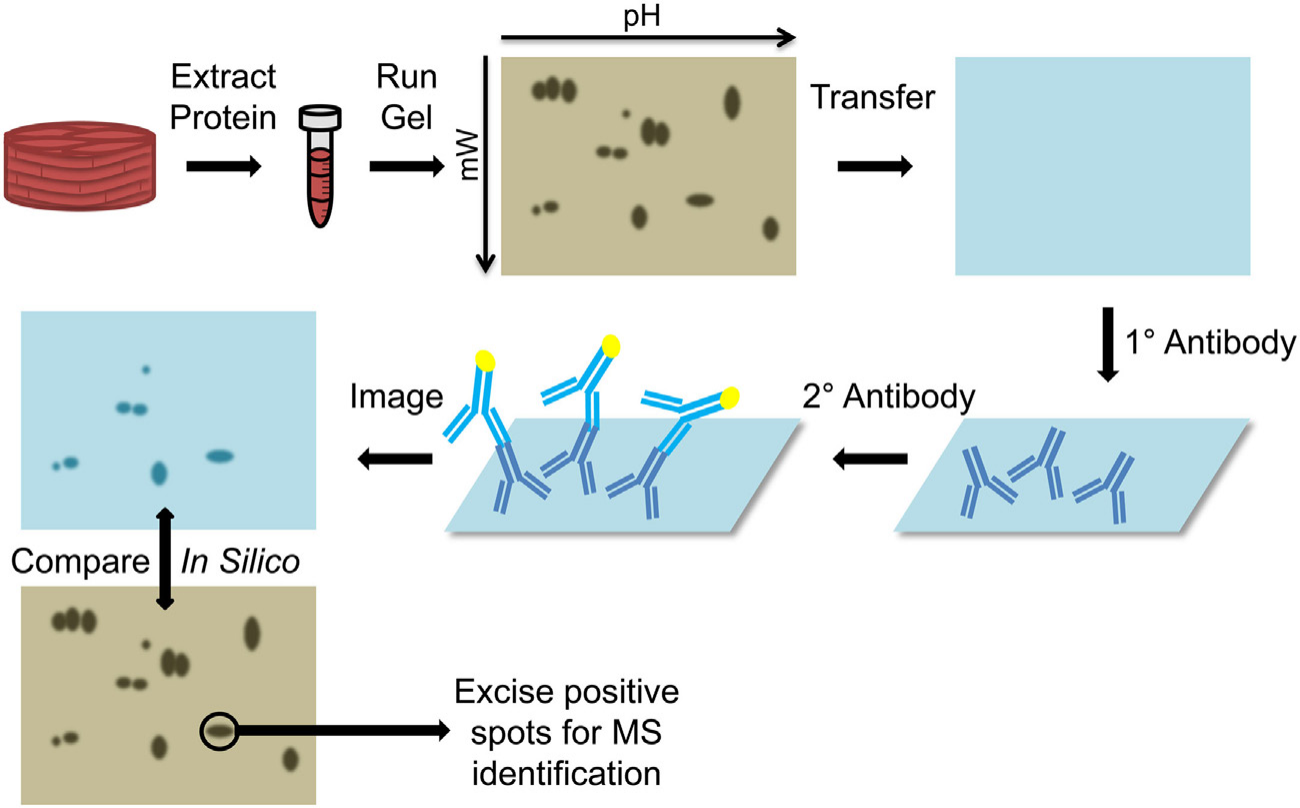
Diagram of two-dimensional Western blots. Protein is extracted from the tissue (cardiac muscle biopsy) and run on a two dimensional gel, based on isoelectric focusing on the *x*-axis and molecular weight on the *y*-axis. The proteins are then transferred to a nitrocellulose membrane and probed with the primary patient serum. Presence of bound human antibodies is detected using a reporter secondary antihuman antibody and imaged to determine the location of immunoreactive spots. This membrane is compared *in silico* to a stained gel and the immunoreactive spots on the gel are excised and submitted for mass spectrometry (MS) identification.

Initial attempts to identify immunogenic antigens in cardiac transplant recipients utilized two-dimensional gel electrophoresis (2-DE) immunoproteomic methods (Figure 3). Vimentin (37), cardiac myosin (31), and heat shock protein-60 (HSP-60) (31) were identified in two seminal papers using a 2-DE approach. Latif et al. extracted protein from human cardiac biopsies and skeletal muscle to test for immunoreactive banding using serum from control subjects with ischemic heart disease and diseased patients with dilated cardiomyopathy (31). The banding pattern from cardiac muscle extracts matched that of skeletal muscle proteins, leading the investigators to suspect a myofibril based antigen. 2-DE-based immunoproteomic analysis of cardiac muscle probed with diseased patient serum identified cardiac myosin and HSP-60 as antigenic (31). Wheeler et al. collected pretransplantation, 12- and 24-month postcardiac transplantation serum, but used cultured endothelial cells as the source of protein for 2-DE Western Blotting (37). This approach identified vimentin as a common immunoreactive antigen. Further confirmation of this finding was achieved by absorbing antivimentin antibodies from the human serum using a slurry of vimentin-Sepharose beads. The antivimentin antibody-depleted serum was used to probe 1-DE Western blots of endothelial cell proteins. Depletion of antivimentin antibodies resulted in marked reduction in reactivity toward bands that corresponded to vimentin’s molecular weight. These publications provided critical proof of concept for identification of cardiac allograft antigenic determinants and prompted numerous investigations into how preexisting or *de novo* recipient non-HLA antibodies affect transplanted hearts (38–41).

Antigen identification using 2-DE immunoproteomic methods relies on three critical aspects of the experimental procedure: (1) all potential antigens are efficiently extracted from cardiac tissue; (2) all extracted proteins can be resolved using a 2-DE format; (3) epitopes recognized by antibodies in patient serum are unaltered by the 2-DE Western blot process. The technical challenges associated with each of these critical steps might explain the generally underwhelming discovery history using 2-DE immunoproteomic methods. In terms of antigen extraction, the majority of reported cardiac antigen identification experiments have relied on a single extraction process (31, 37). The diversity of protein solubility’s make it extremely unlikely that all potential antigens are extracted in a single solution. Indeed, specialized approaches have been developed for membrane (42, 43) and extracellular matrix proteins (44) because their extraction and subsequent proteomic profiling has proven difficult, particularly with 2-DE gel separations. Given that the extracellular matrix and cell membrane of a solid organ transplant are major interfaces between the graft and recipient’s immune system, it is likely that many candidate antigens reside in these subcellular compartments. Indeed, as already discussed, AT_1_R and ET_A_R, membrane receptor proteins, have already been implicated as a cardiac non-HLAs (34). When considering the ability of 2-DE approaches to resolve extracted proteins; the recent realization that a single 2-DE gel spot may actually contain multiple proteins is very concerning (45). This is particularly problematic in immunoproteomic studies, where colocalization of proteins to a single spot has potential to result in attributing antigenicity incorrectly (46). Lastly, the second dimension of the 2-DE process requires that proteins have their disulfide bonds broken and capped, which may result in destruction of conformational epitopes and disruption of linear epitopes. Although studies have demonstrated that linear epitopes can reform following Western Blotting, the ability of conformational epitopes to refold under such conditions is significantly less likely (47). Consequently, even with successful extraction, resolution and blotting, antigen identification may fail since alterations in epitope structure induced by the 2-DE Western blot process prevent antibody recognition. Beyond these issues, 2-DE immunoblotting is challenging to apply to large patient cohorts or multiple tissue types, due to the varying solubility profile of proteins from different tissues, challenging reproducibility of the technique and time consuming workflow. Consequently, despite the first successful application of 2-DE immunoproteomic approach to cardiac antigen identification in 1993, the pool of known non-HLA cardiac antigens has expanded slowly (31). Despite the limitations and challenges of 2-DE immunoproteomics, this approach still remains the most widely utilized method for identification of non-HLAs in cardiac transplant patients and other disciplines (46, 48–50). However, the limitations of 2-DE have led to efforts to develop other methods for immunoproteomic antigen identification.

## Current Antigen Discovery Technologies

The challenges associated with 2-DE immunoproteomic methods prompted development of a number of other antigen identification methods. An ideal antigen identification method should be sensitive, specific, highly reproducible, have high-throughput capabilities, identify antigens in their native configuration, and be applicable for antigens across the entire range of subcellular compartments. Although current technologies fulfill some of these criteria, an ideal immunoproteomic antigen identification method has yet to be developed.

### Protein Microarray

**Figure 4:**
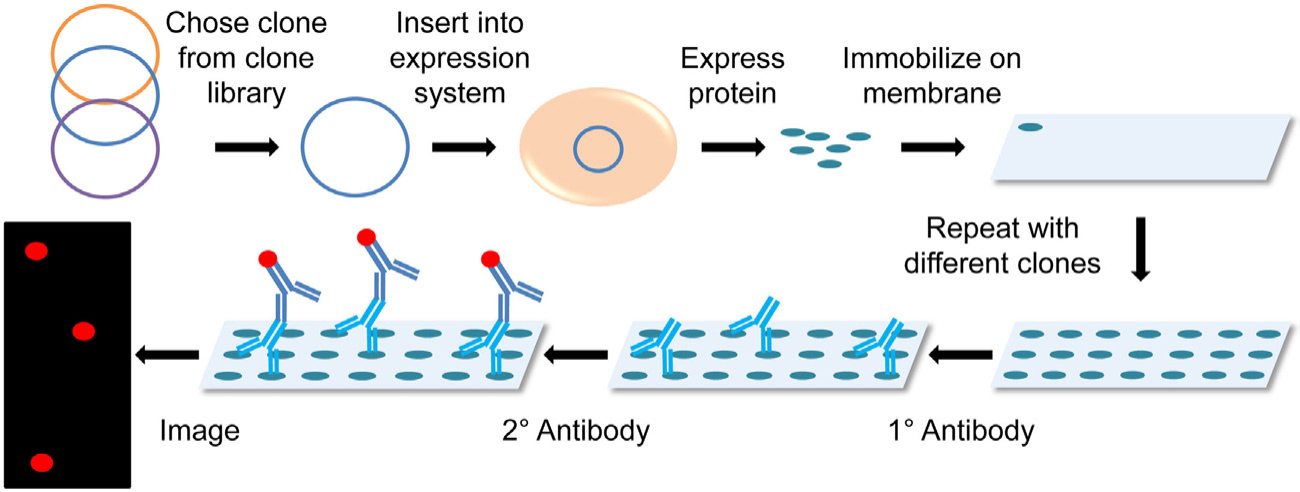
Diagram of protein microarray. Clones are chosen from a clone library and then inserted into an expression system that mass produces the chosen proteins in non-denaturing conditions. These proteins are then immobilized onto a membrane or microscope slide. The membrane is probed with the primary patient serum and detected using a fluorescently labeled secondary antihuman antibody. The membrane is then imaged in a microarray detection system.

ProteoArray^®^ is the most popular microarray utilized in transplant immunology, which pulls proteins from the Ultimate™ ORF clone collection, expresses them using the baculovirus-based expression system and immobilizes the proteins onto a nitrocellulose-coated slide. The slide can then be immunoblotted with patient serum followed by a fluorescent reporter secondary antibody, and read using a multiplex detection system (Figure 4) (51). Research conducted using protein arrays requires pre- and posttransplant serum from transplant patients, with the option to follow-up with ELISAs to further investigate individual identified antigens. So far this technology has only been applied to renal transplant patients (28, 29, 52, 53), but is also popular in other antigen identification applications (e.g., tumor antigens) (51, 54).

While new renal non-HLA targets have been found using protein microarray techniques, none of the experiments identified previously known renal non-HLA targets such as AT_1_R (28, 29, 52, 53). However, researchers did not clarify if these proteins had been purposefully omitted from their microarray analysis. Li et al. (29) did specifically look at HLA and MICA with protein microarray, but were restricted in their search since ProtoArray^®^ only displays four HLA proteins (HLA-B, HLA-DPA1, HLA-DMA, and HLA-DRA). When comparing ProtoArray^®^ to current clinically utilized gold standard solid phase techniques for identification of *de novo* DSA (29), the author’s demonstrated sensitivity of only 56%, but 100% specificity. Additionally, the study found that 72% of patients had an increase in anti-MICA antibodies after transplantation. However, correlation between anti-MICA antibody development and recipient rejection episodes were not reported as part of the study, even though there is evidence that anti-MICA antibodies increase the risk of rejection (55, 56). Dinavahi et al. (52) observed that only one [peroxisomal-trans-2-enoyl-coA-reductase (PECR)] of the three confirmed antigens (PECR, serine threonine kinase 6, peptidyl-prolyl-isomerase-A) expressed on the array was shared between their study patients. This finding led the authors to echo the concerns of Porcherary et al. (28), that the combination of graft-specific non-HLA antibodies produced by each individual may be specific to that individual and not informative of the population as a whole.

Although protein microarrays have been successfully utilized for antigen identification, this approach has important limitations which restrict its overall applicability. Protein microarrays overcome issues of reproducibility associated with 2-DE approaches and are high-throughput methods with over 9,000 proteins printed on a single array. However, proteins printed onto a microarray are not tissue specific, since proteins of interest are chosen from a clone library. This lack of tissue specificity may be critical in transplantation research, where organ dysfunction due to immune activation is of paramount concern. Consequently, lack of tissue specificity requires follow-up steps to confirm that putative antigens are in fact highly expressed in the tissue of interest (29). Additionally, since the entire proteome is unable to be printed on a single chip, proteins of interest have to be selected prior to chip production, which can lead to selection bias and/or incomplete screening of potential antigens. Printing of expressed proteins also creates unique potential complications, including requirement to achieve native protein folding, include posttranslational modifications and difficulty in expression of proteins from the full spectrum of subcellular locations. The baculovirus-based expression system used by popular protein microarrays like ProtoArray^®^ has many advantages, which overcome many of these concerns. The N-terminal glutathione S-transferase-tag system allows for increased expression and purification of protein, generation of cytoplasmic and secreted proteins, stable disulfide bonds within proteins, and the majority of posttranslational modifications are conserved compared to mammalian cells (57–59). However, the predominant drawback of this expression system is that membrane-associated proteins are underrepresented (60). Given the fact that several non-HLAs are membrane receptors, this limitation poses a significant problem when applied to transplantation antigen identification studies (34). The final and potentially most important challenge for the use of microarrays is their expense. With an average array costing approximately $2,000, plus the cost of downstream data analysis, the high cost of each protein microarray prevents widespread application of this technology to large populations or longitudinal studies (52). Consequently, due to the cost and limited number of expressed proteins per slide, protein microarray’s are likely to remain most applicable in the initial screening for non-HLA antibody discovery, rather than routine screening of highly polymorphic known antigens such as HLA.

### Serological Analysis of Recombinant cDNA Expression Libraries (SEREX)

**Figure 5:**
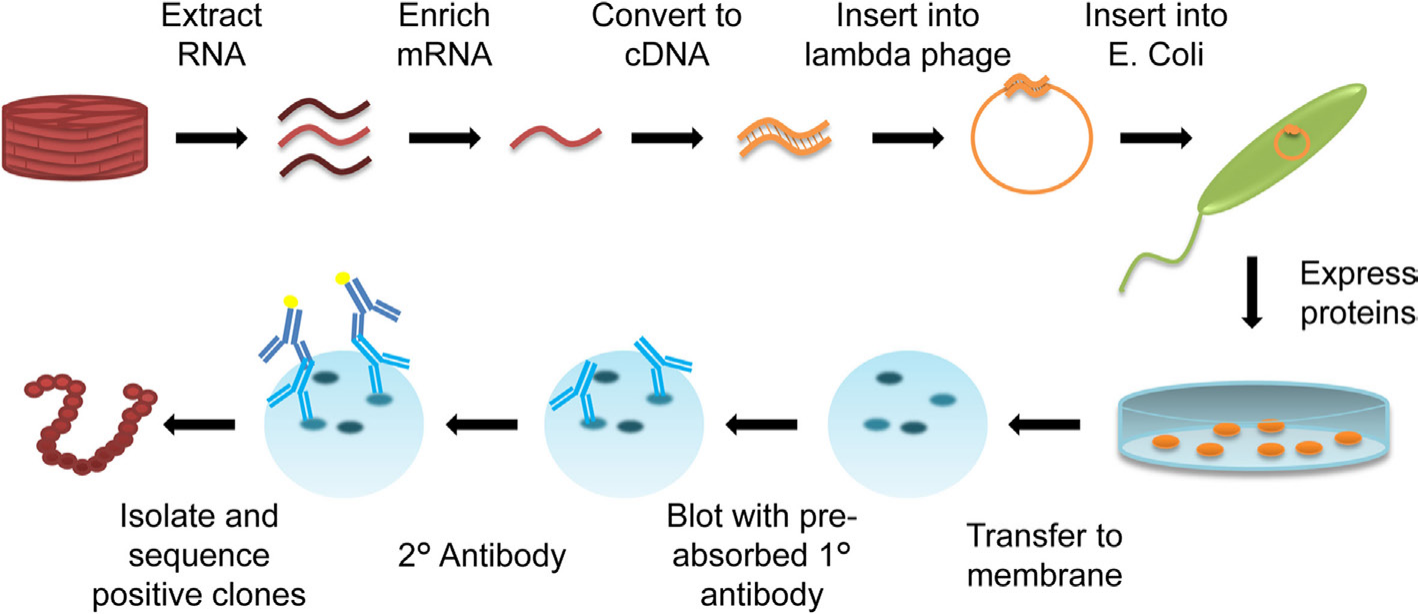
Diagram of serological analysis of recombinant cDNA expression libraries (SEREX). RNA is extracted from organ-specific tissue (cardiac muscle biopsy) and enriched for mRNA. This mRNA is converted into cDNA, and inserted into a λ-phage. The λ-phage expression vector is then transfected into *Escherichia coli*, with each *E. coli* expressing a different protein from the mRNA library on an agar plate. Expressed proteins are transferred to a membrane. Patient serum that has been preabsorbed with *E. coli* proteins to remove any *E. coli*-specific antibodies is used to probe the membrane, and a reporter antihuman secondary antibody is used to visualize immunoreactive colonies. Immunoreactive spots are isolated and sequenced to identify the protein produced by positive clones.

One of the newest techniques applied to transplant immunology is SEREX. Originally developed for cancer antigen identification, SEREX uses patient serum to screen peptide libraries generated from donor organ mRNA. The *Escherichia coli* transfected with donor organ mRNA thereby produce proteins present in the original tissue, with each plaque representing a different protein, which can be probed with patient serum for antigen identification (Figure 5).

To date, the SEREX technique has only been applied to lung transplantation in an attempt to better understand bronchiolitis obliterans syndrome (61). Tissue was harvested from donor trachea and used to build the cDNA library. The resultant SEREX library was then screened with serum from 11 recipients pretransplant and >6 months posttransplant. The resulting data identified six non-HLAs, which were both polymorphic (PSMC4, F3, LOC284058, PLUNC, ZNF33A) and non-polymorphic (XP_931864). However, at most these antigens were only shared between 4 of the 10 patients. These findings demonstrate that the non-HLA antigenic profile of individual patients differ, and consequently more information is needed to fully assess the extent to which each non-HLA results in development of graft-specific antibodies within a particular transplant population.

Unlike protein microarrays, SEREX technology allows for high-throughput screening of tissue-specific proteins. However, one of the major drawbacks of the SEREX approach is that the bacterial expression system used can never create all of the same tertiary protein structures of eukaryotic cells, especially disulfide bonds (62). This limitation leads to the potential for inappropriate protein folding and resultant failure of conformational epitope identification. As noted by the authors, due to the bacterial expression system, SEREX favors identification of antigens in which native protein folding is achieved under basic conditions (61). Finally, SEREX can only identify antigens which stimulate high antibodies titers, which results in potential for important non-HLAs to go undiscovered (61). Although SEREX has been successfully applied to a transplant scenario, the limitations of the technique may mean that it is better suited for its original intent of cancer antigen identification.

### Immunoprecipitation

**Figure 6:**
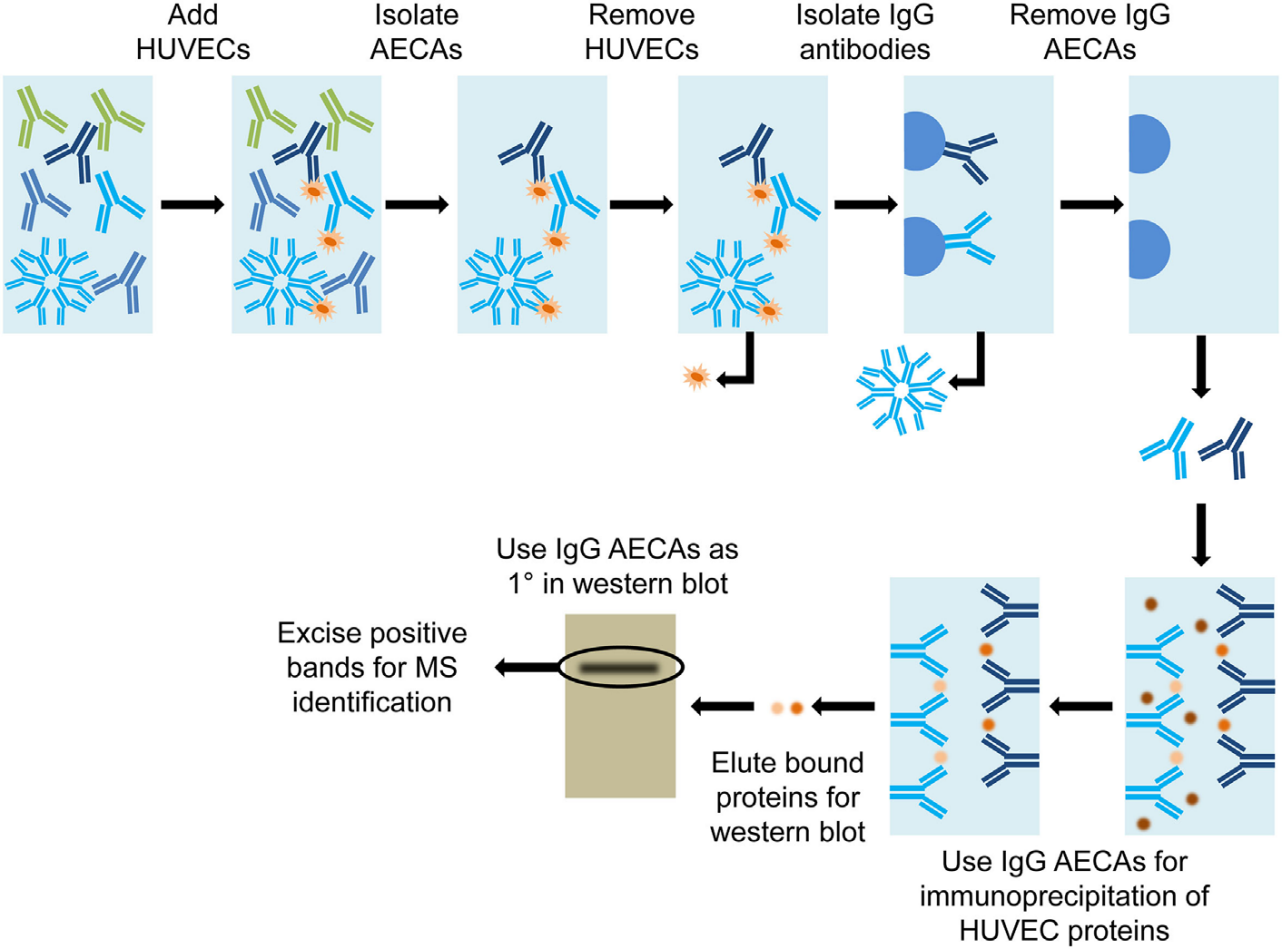
Diagram of immunoprecipitation. Serum from a transplant patient was used to bind human umbilical vein endothelial cells (HUVEC) to achieve affinity purification of antiendothelial cell antibodies (AECAs). HUVECs are washed from the AECAs, to complete the AECA isolation. The IgG-specific AECAs were isolated by binding to Protein A Sepharose beads, followed by collection from the column. Isolated IgG AECAs were then used for immunoprecipitation of HUVEC proteins. The bound proteins were eluted off of the AECAs and run on a Western blot. The IgG AECAs were used again to probe the Western blot and positive bands were excised and submitted for mass spectrometry (MS) identification.

One research group, Qin et al., used a combination of immunoprecipitation and mass spectrometry (MS) (63) to identify antiendothelial cell antibodies (AECAs) in renal and cardiac transplant recipients. Isolated IgG AECAs were used for immunoprecipitation of human umbilical vein endothelial cell (HUVEC) protein extracts, which were run on a Western blot. A single band, appearing on the transplant recipient Western blot that was absent from the normal human Western blot, was excised and submitted for MS identification (Figure 6).

Qin et al. was successful in identifying a single potential antigen, nucleolin, *via* their method. The process was initially conducted with serum from a single transplant recipient. The authors then created a human nucleolin ELISA to assess serum from kidney and heart transplant recipients. Critically, the authors demonstrated that antibodies toward nucleolin correlated with an increased risk of rejection. For instance, 63.9% of heart transplant recipients with antinucleolin antibodies developed transplant-related coronary artery disease, versus only 31.8% of recipients without antinucleolin antibodies. Finally, nucleolin was shown to be a surface protein on endothelial cells, and antinucleolin antibodies inhibited proliferation of endothelial cells. Consequently, the authors demonstrated not only that nucleolin represents an important non-HLA but also that the pathogenesis of antinucleolin antibody production may be related to inhibition of endothelial cell function.

A major drawback of this immunoprecipitation approach is that only one patient’s serum was used for the initial screening process. Given that several research groups have proposed that AECAs may be patient specific, this automatically limits this method’s ability to identify multiple antigenic targets. Additionally, since only a one dimensional separation of HUVEC proteins was undertaken, the risk of having multiple proteins localizing to the same band in the gel was dramatically increased. While HUVECs are a readily available source of endothelial proteins, endothelial cells are known to be very heterogeneous (64) and therefore it is likely that HUVECs would not express the same protein complement as cardiac endothelial cells. These limitations bring into question the global applicability of the Qin et al. method for identification of antigenic proteins in transplant patients. However, it is important to note that because they followed up their initial identification with an ELISA, they validated the association between the antinucleolin antibodies and negative patient outcomes. This secondary validation step will most likely become more critical with time as more antibodies are discovered to ensure relevance to patient populations.

## Known Antigen Screening Technologies

As shown above, most antigen-discovery technologies are time consuming and expensive which generally prohibits their use for routine screening. Consequently, following initial antigen identification, many of the research groups discussed use follow-up tests that are faster and more efficient for screening large population groups. Here, we will discuss the most common techniques for population screening toward known antigens.

### Enzyme-Linked Immunosorbent Assay

**Figure 7:**
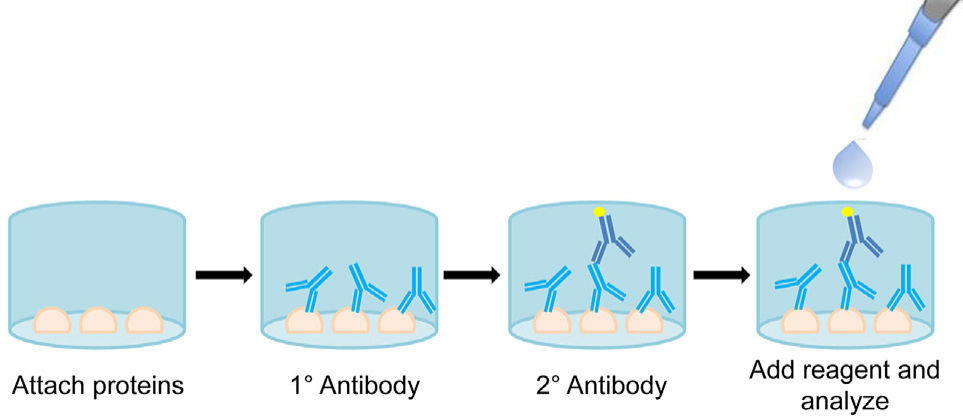
Diagram of enzyme-linked immunosorbent assay (ELISA). Proteins are attached to the bottom of a well and probed with primary patient serum. Bound antibodies are then visualized using an antihuman enzyme conjugated secondary antibody. Reagent signal is proportional to amount of bound antibody, allowing for quantification of antibody titer.

In terms of routine monitoring, ELISAs are a universal standard. OneLambda currently makes ELISAs for anti-AT_1_R or anti-ET_A_R antibodies for screening renal transplant patients. Once optimized, ELISAs provide a rapid, convenient method for bulk screening of patient serum for the presence of non-HLA antibodies (Figure 7). These assays are commonly multiplexed up to 384-well plates and can offer information about antibody presence and individual patient titer. Currently ELISA is the fastest and most efficient method for testing single non-HLA antibodies.

To determine the predictive value of anti-AT_1_R antibodies and their interaction with anti-HLA responses, Reinsmoen et al. used an AT_1_R ELISA assays in conjunction with a Luminex-based SAB assay (i.e., HLA) (23). Serum from 200 patients pre- and postcardiac transplant was utilized for both AT_1_R ELISA and DSA determination. The results indicated a significant increase in the hazard ratio from 7.1 in patients with DSA alone developing AMR and/or CMR compared to 10.5 in patients with both DSA and AT_1_R antibodies. These findings coincide with a common theme of non-HLA antibodies increasing the incidence and severity of rejection when associated with DSA. Undine et al. employed both AT_1_R and ET_A_R ELISAs to investigate the role of non-HLA antibodies in 37 patients receiving intestinal and multivisceral transplantation. The rate of allograft rejection increased from 50 to 80% when the patients developed non-HLA antibodies toward AT_1_R, specifically AMR increased from 11 to 55%. Importantly, the investigators showed that rising titer of non-HLA antibodies coincided with onset of rejection. It is clear that ELISAs for known non-polymorphic antigens have great utility in assessing the timeframe of graft-specific non-HLA antibody development and determining the influence of individual antigens on rejection responses.

Although ELISA methods offer reliable assessment of known antigens, their specificity to a single antigen limits their overall scope. ELISA validation is challenging and frequently associated with a protracted development timeline. Additionally, results vary between laboratories conducting the same ELISA, which can prove to be troublesome and may necessitate development of laboratory-specific reference ranges. Given that the combined presence of non-HLA antibodies and HLA antibodies increases rejection risk, it is entirely possible that presence of multiple non-HLA antibodies may also be found to increase hazard ratio for rejection episodes. Unfortunately, the specificity of ELISA for single antigens makes it impossible to know whether additional non-HLA antibody responses have developed alongside the antibody that is currently being tested. Studies have already shown that there are early and late stage non-HLA antibodies (26), and it is becoming increasingly clear that a matrix (multiple antigen) biomarker approach is likely to be necessary for prediction of rejection episodes. Consequently, although ELISA provides a powerful tool for monitoring graft-specific antibody titer toward known antigens, its limitations may hinder its use in understanding the complexity of the rejection response in individual patients.

### XM-ONE™

**Figure 8:**
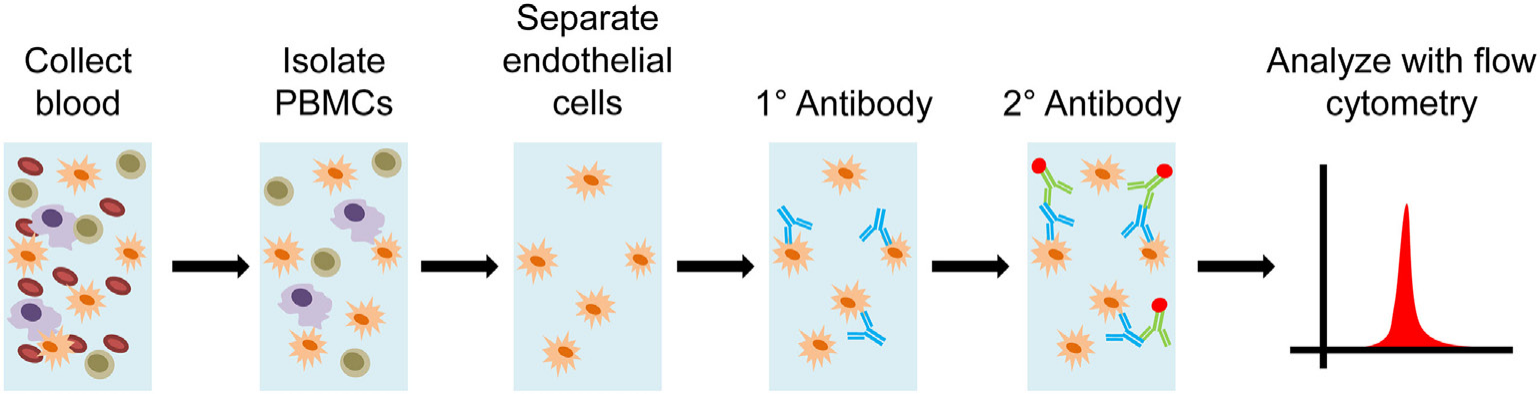
Diagram of XM-ONE assay. Blood is collected from the donor and peripheral blood mononuclear cells (PBMCs) are isolated. Endothelial precursor cells are then isolated from these PBMCs. These precursors cells are then probed by recipient antibodies. The recipient antibodies are then tagged with fluorescently labeled antihuman IgG or IgM antibodies. The antibody bound precursor cells are run through a flow cytometer and patient reactivity toward donor endothelial antigens is determined.

The proprietary XM-ONE™ technology applies the principles of solid phase HLA cross-matching to identification of non-HLA antibodies, *via* screening of AECA. Just as with solid-phase HLA screening, MFI can be used to infer severity of response for individual patients (Figure 8). XM-ONE™ testing in clinical practice has unfortunately met with variable success (65, 66). The company indicates that a positive XM-ONE™ readout correlates with a higher risk for rejection. Although only 24% of tested patients were positive, 46% of positive patients had a rejection within 3 months versus only 12% of negative patients (67). However, a prospective study by Zitzner et al. found that the assay was not predictive of negative graft outcome after 1 year (66). Both studies prospectively enrolled kidney transplant patients, with similar numbers (147 vs. 150), the major difference was that Breimer et al. had multicenter enrollment (67) versus a single transplant center with Zitzner et al. (66) These differing results require future clinical studies to further validate and understand the utility of XM-ONE™ technology in prediction of transplant rejection episodes.

The variable results in XM-ONE™ trials may prove to be troublesome, as opposing reports from different study sites may indicate inherent variability within the way the test is conducted. For instance, given that this is a flow cytometry-based system, there can be bias originating from gating strategies employed at different sites, which may be difficult to standardize. Furthermore, major downside to this technology is that it never identifies specific non-HLAs against which antibodies have been formed. This could mean that important fluctuations within different antigen-specific antibodies can go undetected, as well as providing little additional information about the pathogenesis of rejection. Additionally, since the substrate for the test is precursor endothelial cells, they may not express all of the potential antigens present in transplanted tissue. This concern is further complicated by the fact that endothelial cells themselves express different surface markers depending on their activation state and vessel of origin (64). Finally, since the technique utilizes whole cells, only surface proteins can be detected, leading to the possibility of failing to detect important integral membrane, nuclear, cytoplasmic, or matrix antigens. However, if testing can become consistent between testing facilities then the XM-ONE™ approach may provide clinically relevant information regarding AECA in transplant recipients.

## Future Directions

Work on non-HLAs been ongoing for the last 20 years, with some antigens found in the early 1990s using techniques that are now considered antiquated. It is clear that this work has been successful in identifying a number of non-HLAs capable of influencing rejection incidence and severity. However as previously discussed, current antigen identification methods are far from ideal. Limitations within each method confine the range of proteins available for antigenic screening and curb the pace of identification. Given that each method has its own set of pros and cons, which result in a defined susceptibility to produce false positives and negatives, special care must be taken to ensure that once a target has been found, it is thoroughly validated using other methods (e.g., ELISA). Cardiac myosin was initially found to be antigenic in the context of dilated cardiomyopathy for instance, not transplantation. Later studies using myosin ELISA and ELISPOT demonstrated that myosin is an important autoantigen in cardiac transplants (68). As the pace of antigen identification increases, such validation attempts are likely to be complicated by the fact that the population distribution of many non-HLAs is far from ubiquitous. As the field moves toward high-throughput screening assays using arrays of newly discovered non-HLAs, it is important to consider how the initial identification was achieved, and what validation steps have been taken to confirm identity of each putative antigen.

Technological advances in antigen identification methods offer the possibility of multiplexing of samples, allowing for longitudinal analyses in individual patients and assessment of antigen frequency in large patient populations. Much of the research presented here relied on a comparison of small cohorts with single pre/posttransplant time points (29, 52) or matched cases (28). Our lab has recently validated a novel immunoproteomic method for antigen discovery which is fast, simple and inexpensive compared to the currently available technology (69). It has been applied to an animal model of xenotransplantation and is currently being applied to cardiac transplant patients (69). Although further clinical validation is necessary, this affinity chromatography antigen identification approach has potential to further enhance understanding of recipient non-HLA graft-specific immune responses. Such high-throughput antigen identification methods offer the potential for screening large numbers of patients at multiple postoperative time points. Such approaches are likely to be critical in determining correlations between timing of non-HLA antibody production and clinical symptoms in large cohorts of patients. Understanding the longitudinal graft-specific immune response is critically important to distinguish which antigens are initiators of graft-specific immune response (i.e., primary antigens) and which are simply secondary bystanders of a more generalized non-specific immune activation or break in tolerance resulting from graft damage (i.e., secondary antigens). Ultimately, combining information regarding the distribution of graft-specific antigen response within the population and time course of such responses (i.e., primary vs. secondary antigens) will be critical for development of effective biomarker panels for rejection risk stratification.

## Conclusion

Just as routine DSA screening is not 100% predictive of rejection, it is unlikely that a single non-HLA antibody will be 100% predictive of rejection. It is evident that non-HLA antibodies play an important role in the pathogenesis of cardiac allograft rejection, but to specifically identify their targets has proven to be time-consuming and troublesome. Even with new technologies showing promise, the relative contributions that non-HLA antibodies play in graft destruction is still unknown. Such information represents a critical linchpin to not only understanding but predicting rejection. Clearly this work requires a broader understanding of population responses, and as such, will most likely move toward a more complete biomarker panel vs. a singular “silver-bullet” solution. Ultimately as technology makes its inevitable march forward, techniques that can identify and monitor new antigen targets will develop and improve, along with a deeper understanding of the immunological mechanisms that govern cardiac allograft transplants.

## Author Contributions

KG, LG, and NP were equally involved in the design and conception of this review. KG contributed to the drafting and refinement of the manuscript with the assistance and oversight of LG and NP, who gave final approval of the version to be published.

## Conflict of Interest Statement

The authors declare that the research was conducted in the absence of any commercial or financial relationships that could be construed as a potential conflict of interest.
